# Constitutive Model and Recrystallization Mechanism of Mg-8.7Gd-4.18Y-0.42Zr Magnesium Alloy during Hot Deformation

**DOI:** 10.3390/ma15113914

**Published:** 2022-05-31

**Authors:** Ling Zhang, Xiaoyu Wu, Xiaofeng Zhang, Xindong Yang, Yinglong Li

**Affiliations:** 1 School of Materials Science and Engineering, Northeastern University, Shenyang 110819, China; zlcljg239@163.com (L.Z.); 18842562873@139.com (X.W.); 15640623271@163.com (X.Z.); y_xindong@163.com (X.Y.); 2 School of Mechanical and Engineering, Ningxia Institute of Technology, Shizuishan 753000, China

**Keywords:** Mg-8.7Gd-4.18Y-0.42Zr (GW94K) magnesium alloy, uniaxial compression, constitutive relation, processing map, dynamic recrystallization, rare-earth

## Abstract

The hot deformation behavior of Mg-8.7Gd-4.18Y-0.42Zr alloy was investigated by uniaxial hot compression tests at 300–475 °C with strain rates of 0.002–10 s^−1^. The average activation energy was calculated as 227.67 KJ/mol and a constitutive relation based on the Arrhenius equation was established in this study. The results show that Mg-8.7Gd-4.18Y-0.42Zr magnesium alloy is a strain rate and temperature-sensitive material. When the temperature is constant, the flow stress increases with the increase of strain rate, while when the strain rate is stable, the flow stress decreases with the increase of temperature. DRX is the main softening mechanism of the alloy, including continuous dynamic recrystallization (CDRX) and discontinuous dynamic recrystallization (DDRX). Meanwhile, the DRX grains nucleate preferentially at the twin intersections in the parent grains under the deformation condition below 300 °C and gradually expand outward with the increase of strain. When the compression temperature is above 400 °C, DRX grains nucleate preferentially at the original grain boundary and then gradually expand inward with the increase of strain. The optimum deformation conditions of the studied alloy are performed at 400–450 °C and 0.002–0.02 s^−1^ by a comprehensive comparison of the hot processing map, microstructure refinement, and formability.

## 1. Introduction

Magnesium alloy, as the lightest metal alloy structural material, has been widely concerned in the industrial and scientific community [1,2]. It has been shown that wrought magnesium alloy has higher strength and ductility than cast magnesium alloy, which is beneficial to the further popularization of magnesium alloy [3]. However, they are limited by their poor formability at low temperatures (<200 °C), low strength, and heat resistance [4]. In contrast to conventional magnesium alloys, the magnesium-containing rare-earth elements (RE) have been widely investigated with the aim of developing high strength magnesium alloys due to RE can weaken the texture and effectively enhance heat resistance and corrosion resistance [5,6]. Particularly Mg-Gd-Y systems have excellent comprehensive properties, such as room and elevated temperature strength, corrosion resistance, etc., and have been widely utilized by aerospace, military, and automobile applications [7].

Thermo–mechanical processes are indispensable parts of producing structural components with good mechanical properties and complex shapes because they can eliminate casting porosity in castings and refine the grains. Moreover, the hot deformation behavior of magnesium alloys containing rare earth has always been a hot topic worldwide. In addition, with the rapid development of China’s aerospace and automobile industry, the requirement of energy conservation and emission reduction for aerospace and automobile development is becoming increasingly urgent, therefore, the development of elevated temperature workability of Mg-Gd-Y magnesium alloy has great potential. Exploring the rheological behavior of metals or alloys during hot deformation can provide the theoretical basis for studying their hot workability (such as rolling, forging, extrusion, etc.). The rheological behavior of metal materials is usually described by the relationship between the flow stress produced by thermal activation, the strain rate, and deformation temperature. However, the processing map constructed based on the dynamic material model (DDM) is of extreme significance for understanding the hot workability and finding out the optimum hot working parameters of some materials that are difficult-to-deform [8,9]. Indeed, the Mg-8.7Gd-4.18Y-0.42Zr (GW94K) alloy studied in this research is a typical hard-to-deform material. In the previous study, Zhou et al. [10] investigated the deformation behavior of extruded Mg-9.8Gd-2.7Y-0.4Zr Mg alloy. The result showed that the optimum deformation domain and dynamic recrystallization of Mg-9.8Gd-2.7Y-0.4Zr alloy was the deformation temperature of 420–450 °C and the strain rate of 0.01–0.1 s^−1^. Xia et al. [11] studied the deformation behavior, kinetics, and processing map of coarse-grained Mg-Gd-Y-Nd-Zr alloy, which provided the optimum hot working condition as well as dynamic precipitation and dynamic recrystallization. Xiao et al. [12] studied the dynamic precipitation behaviors of Mg-8.3Gd-2.6Y-0.4Zr alloy during hot compression. They reported that the precipitated phase of the equilibrium β phase (Mg_5_(Gd, Y)) was sensitive to the deformation temperature rather than the strain rate. Yang et al. [13]. investigated the plastic deformation and dynamic recrystallization behaviors of Mg-5Gd-4Y-0.5Zn-0.5Zr (GWZ540) alloy. They developed a new method to quantify the progress of dynamic recrystallization in metals from the stress–strain curve and strain hardening data. According to M. Roostaei et al. [14], the deformation activation energy of Mg-3Gd-1Zn (GZ31) alloy is 173.2 kJ/mol, which is large in comparison with the lattice self-diffusion activation energy of magnesium (135.5 kJ/mol). As a consequence of the presence of rare earth elements, gadolinium is important in increasing the deformation resistance at high temperatures. The flow stress of Mg-Gd-Y-Zr alloy during hot deformation was investigated by Mirzadeh [15]. This study quantitatively demonstrated that the rare earth addition significantly improves the heat strength and hence, the creep resistance. This result has been verified in the research of Alizadeh et al. [16]. In the research conducted by Barezban [17], the behavior of newly developed Mg-Gd alloys was studied to gain a better understanding of how Gd affects the mechanical behaviors of magnesium. Therefore, it is of great significance to study the hot deformation behavior and dynamic recrystallization behavior of hard-to-deform magnesium alloys to optimize process parameters and control the microstructure under various process conditions.

This work aims to investigate the hot workability of Mg-8.7Gd-4.18Y-0.42Zr (wt.%) by thermo-mechanical compression tests. The stress–strain behavior was studied for the severe plastic deformation of the studied alloy by using processing map techniques, and the safety and instability domains were established by a processing map. Besides, the microstructural observation was conducted to validate the safe and instability domains in the tested samples, and the recrystallization mechanism during the hot deformation process was investigated. This paper hopes to provide a reference and theoretical basis for the fabrication of high-performance magnesium components containing rare-earth elements.

## 2. Experimental Procedure

Firstly, the master alloys were obtained by using high purity alloying elements Mg (>99.95%), Gd (>99.9%), and Y (>99.9%) in a medium frequency electromagnetic induction vacuum smelting furnace under argon protection. Then Mg-8.7Gd-4.18Y-0.42Zr(wt.%) ingots were obtained by smelting high purity Mg, Mg-25% Gd, Mg-25% Y, and Mg-30% Zr master alloys. This process was carried out in a well-type resistance crucible furnace shielded by SF_6_/CO_2_ gas, and a cylindrical ingot with a diameter of 150 mm and a height of 250 mm was obtained by pouring in a graphite crucible. A square plate 50 mm in length and width and 20 mm in thickness was cut from the middle of the ingot and was solution treated at 500 °C for 10 h, then quenched into hot water at ~60 °C (i.e., T4 treatment). Cylindrical specimens with a diameter of 8 mm and a height of 15 mm were cut from the square plate after T4 for a hot compression test. The actual chemical composition of the alloy ingots was measured by an inductively coupled plasma emission spectrometer (ICP-AES Plasma 2000).

The uniaxial isothermal compression experiment was carried out with the MMS-200 thermal simulator of the State Key Laboratory of Rolling and Automation of Northeastern University. With an automatic data acquisition system installed on a computer, the nominal stress–strain data were continuously monitored during compression. The true stress and true strain were determined from nominal stress and strain data according to the following formulas: *σ_T_* = *σ_N_*(1 + *ε_N_*), *ε_T_* = ln(1 + *ε_N_*), where *σ_T_* and *σ_N_* are true stress and nominal stress, and *ε_T_* and *ε_N_* are the true strain and nominal strain, respectively [18,19]. Before the hot compression experiment, two thermocouple wires were welded at the mid-height of the specimen’s surface for temperature measurement and control, and the specimens were resistance heated to the target temperature (*T*) (300, 350, 400, 450, and 475 °C with a variation of ±3 °C) within 1 min and hold for 3 min to obtain a stable and uniformity temperature throughout the specimen. The corresponding strain rates ($\dot{\varepsilon }$) were 0.002 s^−1^, 0.02 s^−1^, 0.2 s^−1^, 2 s^−1^ and 10 s^−1^. 

**Figure 1:**
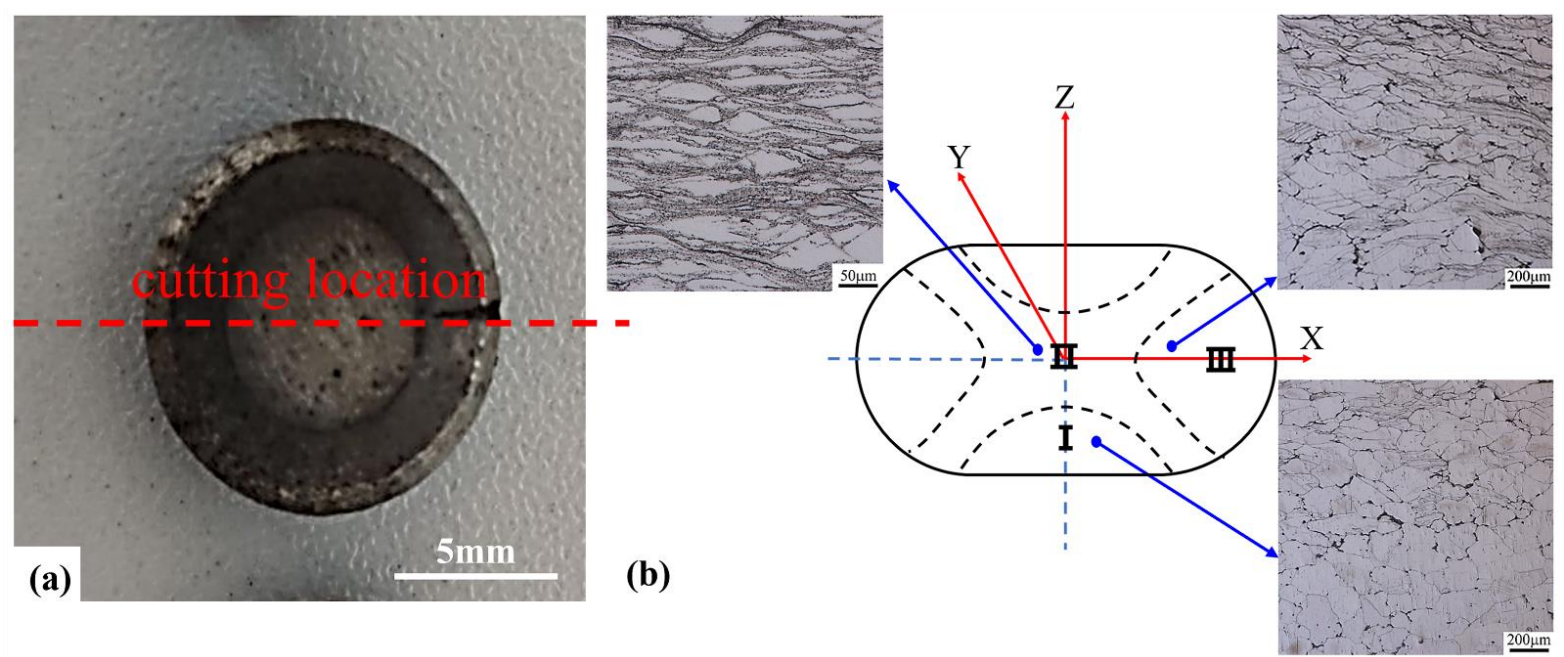
(**a**) Macroscopic photo of compressed sample and (**b**) the microstructures in different deformation domains.

In order to reduce friction, MoS_2_ and graphite lubricant were coated on the end face of the cylindrical sample. The true strain was calculated as *ε* = ln(*h*_0_/*h*_1_), where h_0_ and h_1_ are the initial and final height of samples, respectively. At the end of compression tests, the height was reduced by 66.6% by using the true strain of about *ε* = 1.09. After compression, all the samples were immediately quenched into the water to maintain their deformed microstructure. Each compression test was repeated at least three times with three different samples to ensure the accuracy of the results. Then, the compressed samples were cut along the radial center with a WEDM machine (DK7740B-CG, CQL). The feed speed of molybdenum wire is 0.8 mm/s. The observation position is the geometric center position of the sample, which is more conducive to slip generation and plastic deformation and is an easy deformation zone II, as shown in Figure 1b. Macroscopic photos of the compressed sample and the microstructures in different deformation domains are shown in Figure 1. The cast specimens and compressed samples were mechanically polished with Wuxi brand sandpaper according to the standard procedure, then polished with 0.5 μm diamond grinding paste to make the surfaces of the samples mirror without any scratches. The polished surfaces were etched with a chemically corrosive mixture of picric acid (5.5 g), ethanol (18 mL), water (10 mL), and acetic acid (5 mL), followed by rinsing immediately with ethanol. The microstructures of the samples were observed by optical microscope (OM, Olympus-DSX500).

## 3. Results and Discussion

### 3.1. Microstructure of the As-Solutioned Material

**Figure 2:**
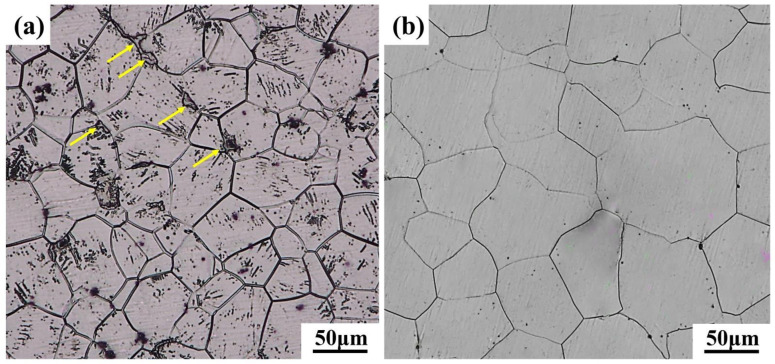
Microstructures of (**a**) as-cast and (**b**) as-solutioned GW94K magnesium alloy.

Figure 2 shows the optical microstructure photos of GW94K magnesium alloy. The as-cast alloy was mainly composed of α magnesium matrix, that is, a solid solution of magnesium containing Gd and Y elements, and a coarse eutectic *β*-phase (Mg_24_(Gd, Y)_5_) [20,21,22], in which the eutectic phase was mainly distributed along grain boundaries as shown by yellow arrows in Figure 2a. After solution treatment (T4) at 500 °C for 10 h, the eutectic structure in the as-cast ingot had completely dissolved into the α-magnesium matrix, as shown in Figure 2b. It was observed that the grains were coarsened obviously, with the average grain size being about 108μm.

### 3.2. True Stress–Strain Curve Characteristics of GW94K Magnesium Alloy

**Figure 3:**
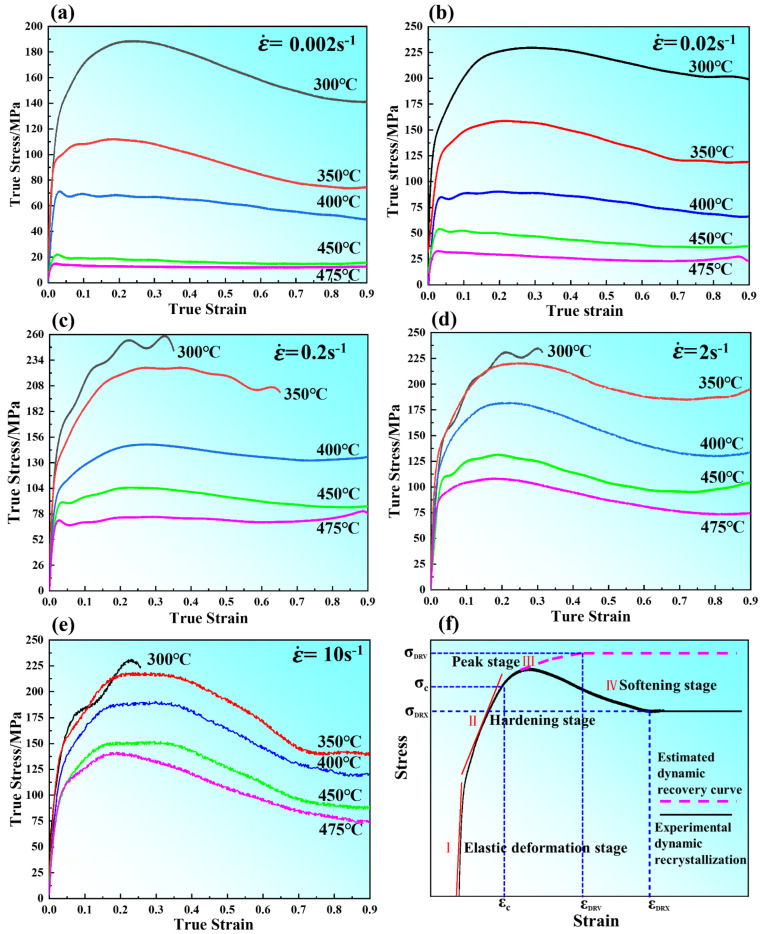
Flow stress curves of the as-solutioned GW94K alloy over the temperature range of 300~475 °C at strain rates of (**a**) 0.002 s^−1^, (**b**) 0.02 s^−1^, (**c**) 0.2 s^−1^, (**d**) 2 s^−1^, (**e**) 10 s^−1^, (**f**) schematic presentation of the flow curves for dynamic recovery and dynamic recrystallization.

Figure 3 shows the true stress–strain curves under different deformation conditions, and the final strain values of the sample are 1.09, which indicates that the alloy had preferable formability. At the initial stage of deformation, the flow stress increased rapidly due to the work hardening caused by the generation and accumulation of many dislocations. With further deformation, dislocations accumulated and entangled to form dislocation walls, which hindered the movement of dislocations and increased the deformation resistance of materials until the flow stress reached the maximum. With the increase of strain, the softening effect caused by dynamic recovery (DRV) and dynamic recrystallization (DRX) made the flow stress decrease slowly, while the temperature rose rapidly and the stress decreased slowly, which was caused by the failure to dissipate the heat generated by plastic deformation in time. Similar stress–strain curves have been observed in other magnesium alloys as well [23,24,25]. At a relatively low strain rate (0.002 s^−1^~0.2 s^−1^) and a relatively high compression temperature (450 °C~475 °C), the flow stress reached its peak and then presented a steady flow (Figure 3a–c), which indicates that the competition between work-hardening and work-softening reached a dynamic equilibrium when the strain exceeded a certain value, under this deformation condition, the work hardening rate decreased with the increase of temperature, indicating that DRX occurred earlier. Moreover, the flow stress curve composed of the work hardening regime and work softening regime showed typical DRX features due to the strong DRX softening and weak strain hardening. However, while at a low temperature (300~350 °C) and a relatively high strain rate, the flow curves exhibited higher peak stress and corresponding peak strain due to the strong strain hardening and weak DRX softening. Therefore, the flow stress exhibited a sharp decrease or premature termination due to fracture, as shown in Figure 3c–e. Table 1 shows the failure strains of the specimens deformed at relatively low compression temperature.

Figure 3f shows the schematic of dynamic recovery (DRV) and dynamic recrystallization (DRX) of materials during the rheological process. During dynamic recrystallization materials, such as close-packed hexagonal materials, with the increase of strain, the flow stress increased due to the occurrence of dynamic recovery (DRV) until the critical strain(*ε_c_*) was reached, at which the generation and annihilation of dislocations offset each other. With further straining, the dynamic recrystallized material exhibited a peak (*σ_p_*) above critical stress (*σ_c_*), at which dynamic recrystallization initiated followed by a work softening process, as shown by the black line in Figure 3f. However, the low stacking fault energy (face-centered cubic) metals reached steady-state stress under critical strain (*ε_DRV_*) during deformation, and a further increase in strain (above *σ_p_*) did not result in a decrease in stress, shown by a pink dotted line in Figure 3f. In this Figure, the curves can be divided into four stages [26]. Stage(I) is the elastic deformation segment; the flow stress increased almost linearly where the sub-grains formed due to dislocation generation and multiplication. Stage (II) with the hardening stage occurred and the curve slope decreased due to the dynamic recovery rate slowing. In stage (III), the effect of DRV was the strongest because the softening effect of DRV cannot be counteracted with the work hardening effect, which leads to the continuous increase of flow stress until the peak stress. Then, in stage (IV) the dynamic recrystallization took place when the strain reached a critical value. With further deformation, the complete dynamic recrystallization occurred.

**Figure 4:**
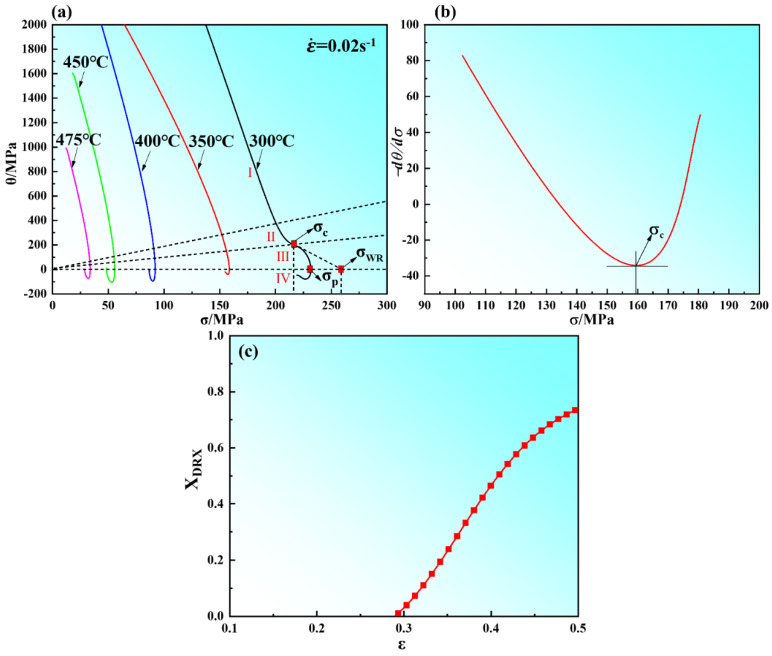
Relationship between *θ* and *σ*: (**a**) *θ*-*σ*, (**b**) -(*dθ*/*dσ*)-*σ*. (**c**) Relationship between X_DRX_ and *ε*.

**Figure 5:**
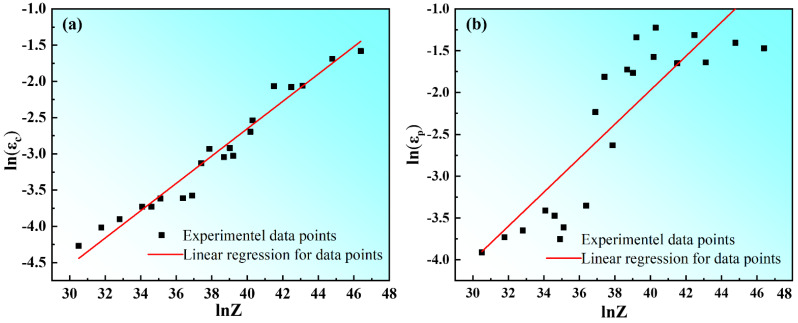
Relationship between critical strain (*ε_c_*) and Zener–Hollomon parameters (Z-parameter) (**a**), and correlation between peak strain (*ε_p_*) and Zener–Hollomon parameters (Z-parameter) (**b**).

The purpose of evaluating the variations of work hardening rate (*θ* = *dσ*/*dε*) and true stress (*σ*) of the material is to accurately determine the true stress and corresponding true strain values at the initiation of DRX. The determination of the beginning of DRX is related to the inflection point of strain hardening rate (*θ*) versus flow stress (*σ*) curve according to abbas NAJAFIZADEH [27] and H. J. McQueen’s [28] method. The relationship between the strain hardening rate (*θ*) and flow stress (*σ*) with a strain rate of 0.02 s^−1^ is shown in Figure 4a. The peak stress value (*σ_p_*) is obtained from the curve at which the strain hardening rate *θ* is zero (in Figure 4a). The stress *σ_WR_* is the steady stress of materials during work hardening and dynamic recovery [29], which is obtained by the extrapolation of the *θ* vs. *σ*. The initiation of the DRX process is expressed by the inflection point (*σ_c_*) of the curve in Figure 4a and the lowest point (*σ_c_*) of the curve in Figure 4b. From Figure 4a, the peak stress decreased with the decrease of strain rate and the increase of compression temperature. This is attributed to the relatively low strain rate can provide enough time for DRV and DRX. At elevated temperatures, DRV and DRX occur relatively fully and the softening effect is enhanced. In this Figure, the curves also can be divided into four stages [30,31]; this is consistent with the results of the analysis in Figure 3f. From the stress–strain curves, the volume fraction of DRX that the specimen is deformed at 0.02 s^−1^ and 450 °C can be calculated and is shown in Figure 4c. Figure 5 shows the relationship between critical strain (*ε_c_*), peak strain (*ε_p_*), and Zener–Hollomon parameters of the Mg–8.7Gd–4.18Y–0.42Zr magnesium alloy. An earlier study [32] indicated that the relationship between the critical strain (*ε_c_*) and the peak strain (*ε_p_*) was in the range of (0.6~0.85) *ε_p_* as the critical strain to express the beginning of dynamic recrystallization. The results in Table 2 show that the ratio of critical strain to peak strain ranged from 0.72 to 0.77, with an average value of 0.74.

**Figure 6:**
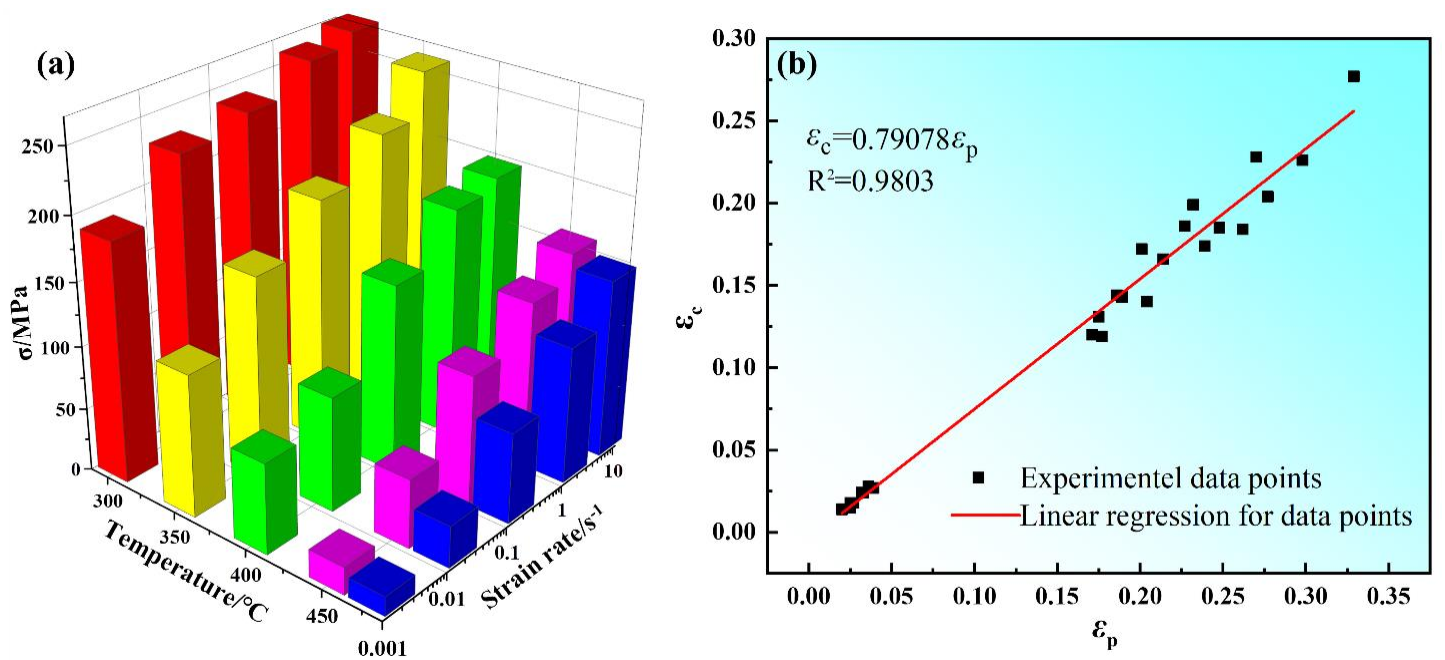
The relationship between peak stress (*σ_p_*), deformation condition (**a**), critical strain (*ε_c_*), and peak strain (*ε_p_*) (**b**).

Figure 6a shows the sensitivity of peak stress of Mg-8.7Gd-4.18Y-0.42Zr magnesium alloy to compression temperature and strain rate. Firstly, when the strain rate was constant, the peak stress decreased with the increase of compression temperature. Secondly, the peak stress increased with the increase in strain rate. This was attributed to the thermal activation energy of materials being low at relatively low compression temperature, and dislocations proliferate and entangle with each other, which leads to the decrease of softening effect caused by dynamic recovery and dynamic recrystallization. The fact that a relatively low strain rate will provide enough time for DRV and DRX. Figure 6b shows the relationship between critical strain (*ε_c_*) and peak strain (*ε_p_*). It is indicated that *ε_c_* has a good linear relationship with *ε_p_*. This is consistent with the earlier studies [33,34]. Through regression analysis, the empirical equations are obtained as follows:

(1) $$\varepsilon_{c}=4.42\times 10^{-5}Z^{0.183}$$

(2) $$\varepsilon_{p}=4.09\times 10^{-5}Z^{0.203}$$

### 3.3. Deformation Action Energy and Constitutive Relation

The constitutive equation can be used to determine the process parameters for the thermo-mechanical deformation of alloys, which can indicate the deformation resistance of materials without the deformation parameters during thermo-mechanical deformation. Compression temperature and strain rate have a significant influence on the variation trend of flow stress and the occurrence of DRX. The relationship between strain rate, flow stress, and compression temperature was proposed by Arrhenius and described by the most common constitutive equation model as follows [24,26,35]:

(3) $$\dot{\varepsilon }=A{\left[\sinh(\alpha \sigma )\right]}^{n}\exp(-\frac{Q}{RT})$$

where$\dot{\;\varepsilon }$ and *T* are strain rate and compression temperature, *R* and *Q* are ideal gas constant (8.31 J/mol) and deformation activation energy, *n* is the stress exponent, *A* and *α* are dimensionless material constants. When *x* is very small, sinh(*x*) approximately is *x,* when *x* is very large, sinh(*x*) about equals *e^x^/*2 by the mathematical analysis. Therefore, when *ασ* < 0.8 and *ασ* > 1.2, respectively, the Formula (3) can be expressed by Equations (4) and (5).

(4) $$\dot{\varepsilon }=A_{1}\sigma^{n_{1}}\exp(-\frac{Q}{RT})$$

(5) $$\dot{\varepsilon }=A_{2}\exp(\beta \sigma )\exp(-\frac{Q}{RT})$$

where *A*_1_, *A*_2_, and *n*_1_ are mathematical constants. Sellars and McTaggart [36] proposed a semi-empirical equation, Based on which the Zener–Holloman parameter expression can be used to describe Equation (3), which indicates the relationship between *Z*, $\dot{\varepsilon }$, and *T*.

(6) $$Z=\dot{\varepsilon }\exp(\frac{Q}{RT})$$

Thus, the expression for *Z* can be obtained from the Equations (1) and (3) and Equations (1) and (6).

(7) $$Z=A{\left[\sinh(\alpha \sigma )\right]}^{n}$$

From Equation (7), the relationship between *σ* and *Z* can be obtained as follows.

(8) $$\sigma =\frac{1}{\alpha }\ln\left\{{\left(\frac{Z}{A}\right)}^{\frac{1}{n}}+{\left[{\left(\frac{Z}{A}\right)}^{\frac{2}{n}}+1\right]}^{\frac{1}{2}}\right\}$$

By taking natural logarithms on both sides of Equations (3)–(5), the following three expressions can be obtained.

(9) $$\ln\dot{\varepsilon }=\ln A+n\ln\left[\sinh(\alpha \sigma )\right]-\frac{Q}{RT}$$

(10) $$\ln\dot{\varepsilon }=\ln A_{1}+n_{1}\ln\sigma -\frac{Q}{RT}$$

(11) $$\ln\dot{\varepsilon }=\ln A_{2}+\beta \sigma -\frac{Q}{RT}$$

The expression of deformation activation energy *Q* was obtained by taking partial differential on both sides of Equations (6) and (7).

(12) $$Q=R{\left\{\frac{\partial \ln\dot{\varepsilon }}{\partial \ln\left[\sinh(\alpha \sigma )\right]}\right\}}_{T}{\left\{\frac{\partial \ln\left[\sinh(\alpha \sigma )\right]}{\partial (\frac{1}{T})}\right\}}_{\dot{\varepsilon }}=RnS$$

**Figure 7:**
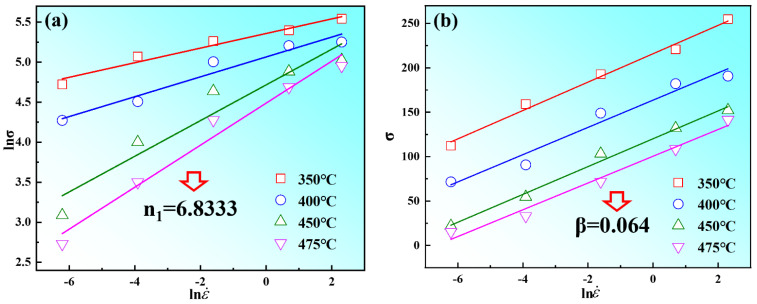
The linear relationship between stress and strain rate: (**a**) the relationship between ln*σ* and ln$\dot{\varepsilon }$; (**b**) the relationship between *σ* and ln$\dot{\varepsilon }$.

**Figure 8:**
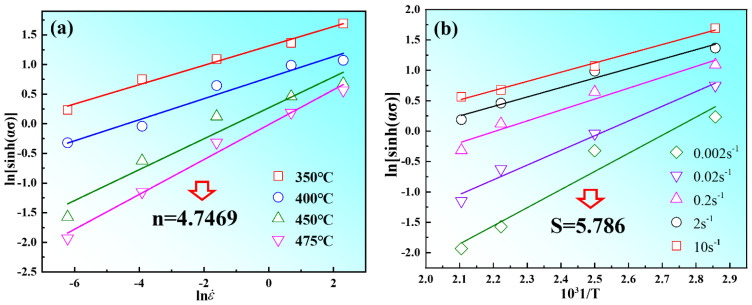
The linear relationship between stress and strain rate: (**a**) the relationship between ln(sinh(*ασ*)) and ln$\dot{\varepsilon }$; (**b**) the relationship between ln(sinh(*ασ*)) and 1/*T*.

According to Equations (9)–(11), *n*_1_ = *∂*ln$\dot{\varepsilon }$/*∂σ*, *α* can be obtained by the ratio of *β* to *n*_1_ (*α = β/n*_1_). The stress *σ* and strain rate $\dot{\varepsilon }$ are substituted into Equations (9)–(11), and the relationships of ln$\dot{\varepsilon }$-ln(sinh(*ασ*)) ln$\dot{\varepsilon }$-ln(*σ*), ln$\dot{\varepsilon }$-*σ*, are linearly fitted, respectively. From Figure 7, there is a good linear fitting relationship between peak stress *σ_p_* and strain rate$\;\dot{\varepsilon }$. The values of *n*_1_ and *β* are calculated as 6.833 and 0.064, according to Figure 7a,b. Therefore, the value of *α* was determined to be 0.0094. In Formula (12), the first item is the average slope (*n*) of 4.74, as shown in Figure 8a; the second term is the average slope (*S*) of 5.78, which is determined by the linear fitting of ln[sinh(*ασ*)]−1/*T*, as shown in Figure 8b. Therefore, the thermal activation energy of Mg-8.7Gd-4.18Y-0.42Zr alloy can be calculated as 227.67 KJ/mol in this experiment.

Equation (13) was acquired by taking natural logarithms of both sides of Equations (6) and (7).

(13) $$\ln Z=\ln\dot{\varepsilon }+\frac{Q}{RT}=\ln A+n\ln[\sinh(\alpha \sigma )]$$

**Figure 9:**
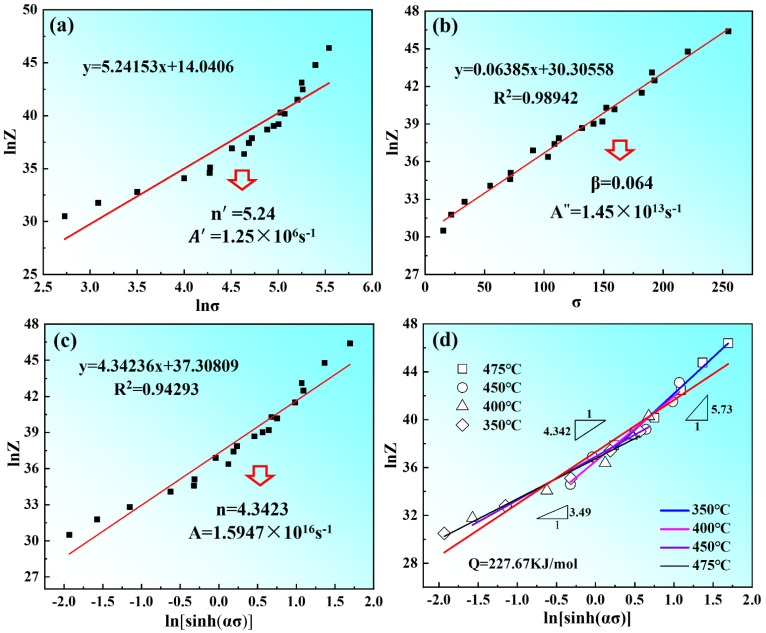
The linear relationships between ln*Z* and ln*σ* (**a**); the relationship between ln*Z* and *σ* (**b**); the relationship between ln*Z* and ln[sinh(*ασ*)] (**c**); linearization of ln*Z*-ln[sinh(*ασ*)] data showing the low-stress region and high-stress region with a stress exponent of 3.49 and 5.73, respectively (**d**).

The values of *Q* and *Z* parameters under different deformation conditions can be obtained according to Equation (13). The linear relationships of ln*Z*-ln*σ* and ln*Z*-*σ* are used to calculate the values of *n*′ and *β* are 5.24 and 0.064, respectively, according to the slope and intercept of the fitting line, as shown in Figure 9a,b. Thus, the values *A*′ of and *A*″ are determined to be 1.25 × 10^6^ and 1.45 × 10^13^. The linear relationship of ln*Z*-ln[sinh(*ασ*)] is used to determine the values of *n* and *A* are 4.34 and 1.59 × 10^16^, respectively, are shown in Figure 9c. When substituting the values of *A* and *n* into Equations (3) and (8), the constitutive equation of the as-solutioned Mg-8.7Gd-4.18Y-0.42Zr alloy can be expressed as:

(14) $$\dot{\varepsilon }=1.59\times 10^{16}[\sinh{\left(0.009\sigma \right)}^{4.34}\exp(-\frac{227.67}{RT})$$

(15) $$\sigma =105.95\times \ln\left\{{\left(\frac{Z}{1.59\times 10^{16}}\right)}^{\frac{1}{4.34}}{\left[{\left(\frac{Z}{1.59\times 10^{16}}\right)}^{\frac{2}{4.34}}+1\right]}^{\frac{1}{2}}\right\}$$

(16) $$Z=\dot{\varepsilon }\exp(\frac{Q}{RT})=\{\begin{array}{c} 1.25\times 10^{16}\sigma_{p}^{5.24}\\ 1.45\times 10^{13}\exp(0.06385\sigma_{p})\\ 1.59\times 10^{16}{\left[\sinh(0.0094\sigma_{p}\right]}^{4.34} \end{array}$$

The result of the linear relationship of ln*Z*-ln[sinh(*ασ*)] is shown in Figure 9d. In general, the stress exponent *n* was utilized to linearize the data, which can be obtained according to the slope of the fitting line in the range of 350 °C~475 °C. It can be found that two lines with a slope of 3.49 and 5.73 indicated that all the data were divided into two regimes, the low-stress regime and the high-stress regime. The dividing point was *σ* equal to 150 MPa and ln*Z* equal to 36. In the low-stress region with a stress exponent of 3.49, the corresponding deformation temperature is in the range of 450–475 °C, which indicates that the creep deformation is controlled by dislocation slip or cross slip. In the high-stress region with a stress exponent of 5.73, the corresponding deformation temperature ranged from 350–400 °C, which indicates that the creep deformation was controlled by dislocation climbing [22,37].

### 3.4. Hot Processing Map of the Mg-8.7Gd-4.18Y-0.42Zr Alloy

It is well known that the hot processing map which is superimposed by the power dissipation map and the instability map in the space of strain rate and compression temperature and can be used to determine the optimum hot deformation parameters. In this study, the hot processing map of Mg-8.7Gd-4.18Y-0.42Zr magnesium alloy was established based on the dynamic material model (*DMM*); that is, the alloy can be considered as a system during the thermo–mechanical process, and the total energy (*P*) obtained from the outside of alloy can be divided into the energy consumed by plastic deformation (*G*) and microstructure transformation (*J*), the expression is written as follows:

(17) $$P=\dot{\varepsilon }\times \sigma =G+J=\int_{0}^{\sigma }\dot{\varepsilon }d\sigma +\int_{0}^{\dot{\varepsilon }}\sigma d\dot{\varepsilon }$$

The strain rate sensitivity index *m* of the alloy is related to the content *G* and *J*, which is determined by the relationship between the flow stress and strain rate. Therefore, the *m* can be written as follows:

(18) $$m=\frac{\partial J}{\partial G}=\frac{\dot{\varepsilon }\partial \sigma }{\sigma \partial \dot{\varepsilon }}=\frac{\partial (\ln\sigma )}{\partial (\ln\dot{\varepsilon })}$$

The dissipative force (*J*) is defined as follows [10]:

(19) $$J=\int_{0}^{\sigma }\dot{\varepsilon }d\sigma =\frac{m}{m+1}d\dot{\varepsilon }$$

where *m* varies in the range of 0 to 1, when *m* = 1, the material is in an ideal linear dissipative state, then *J* reaches the maximum value:

(20) $$J_{\max}=\frac{1}{2}\sigma \dot{\varepsilon }$$

The power dissipation factor is obtained by comparing the Equations (19) and (20):

(21) $$\eta =\frac{J}{J_{\max}}=\frac{2m}{m+1}$$

**Figure 10:**
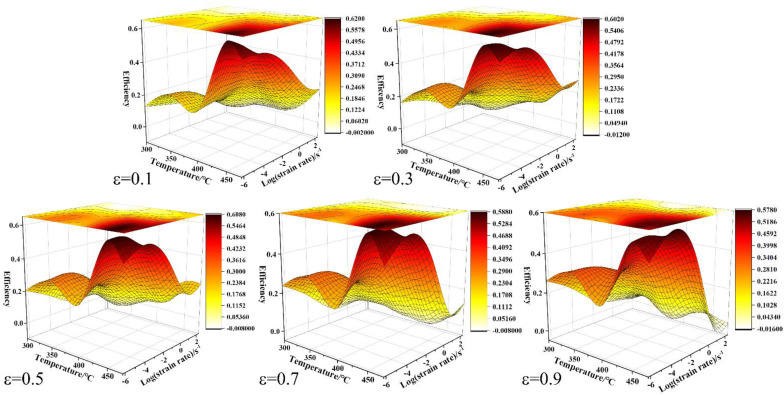
Efficiency maps of power dissipation for the Mg-8.7Gd-4.18Y-0.42Zr alloy at different strains.

The microscopic deformation mechanism of the alloy under the conditions of deformation temperature and strain rate is elucidated. The power dissipation diagram can reflect the variation of *η* with temperature and strain rate. The power dissipation efficiency (*η*) can be calculated and presented by a three-dimensional diagram, as shown in Figure 10. It is found that the relatively dark color represents the region with the highest power dissipation efficiency (*η*) in the power dissipation diagram. Obviously, there are obvious differences between different strains. Firstly, the power dissipation efficiency (*η*) is the lowest under the condition of high strain rate and low deformation temperature. Secondly, when the strain rate is low and the deformation temperature is high, the maximum power dissipation efficiency can be obtained, such as the deformation condition of 450 °C/0.002 s^−1^. Thirdly, the dense contour lines gradually expand to the low temperature and low strain rate region with the increase of strain. It was found that the power dissipation efficiency diagram showed an obvious feature of a relatively dark color at low temperature and a low strain rate when the compression strain was 0.9.

**Figure 11:**
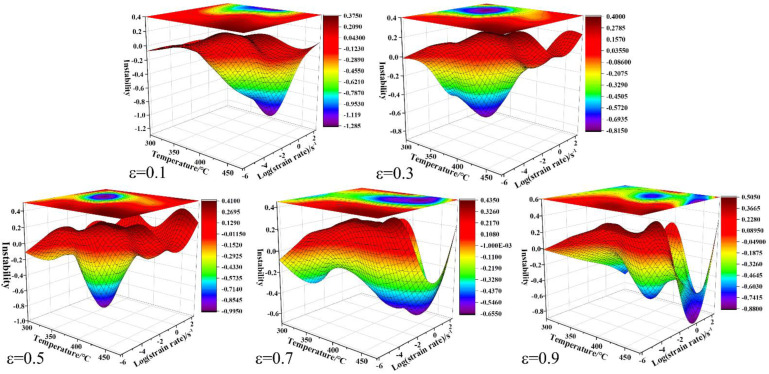
Different instability zones at the different strains.

According to the principle of power dissipation, the inequality of instability condition is expressed as follows:

(22) $$\xi (\dot{\varepsilon })=\frac{\partial \ln(\frac{m}{m+1})}{\partial \ln\dot{\varepsilon }}+m<0$$

where *ξ*($\dot{\varepsilon }$) is the instability parameter. The instability diagram can be used to describe the variation of instability parameter *ξ*($\dot{\varepsilon }$) with temperature and strain rate. The safety condition of hot processing of alloys can be found in the instability map; that is, the instability domain is characterized by *ξ*($\dot{\varepsilon }$) > 0. The instability diagrams obtained at a strain of 0.1, 0.3, 0.5, 0.7, and 0.9 are shown in Figure 11, where the rainbow zones represent the unstable flow and the instability domain featured by negative *ξ*($\dot{\varepsilon }$), while the red zones represent the safe deformation. It was found that the region of unstable flow gradually expanded with the increase of true strain. Especially, the unstable flow expanded to the high strain rate domain when the true strain increased to 0.7 and 0.9.

**Figure 12:**
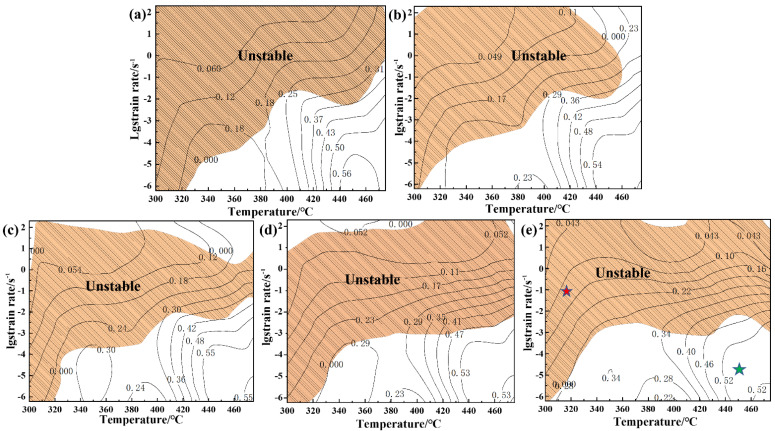
Hot processing map of Mg-8.7Gd-4.18Y-0.42Zr alloy at different strains: (**a**) 0.1, (**b**) 0.3, (**c**) 0.5, (**d**) 0.7, (**e**) 0.9.

**Figure 13:**
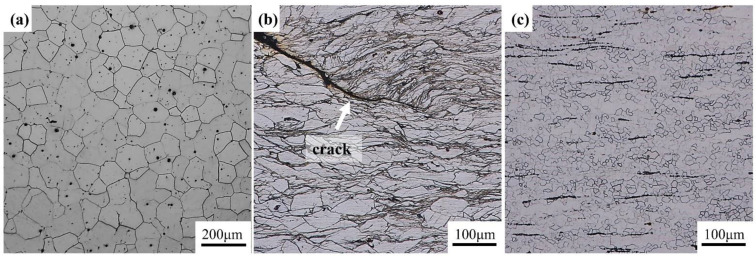
(**a**) initial as-solutioned state, (**b**) instability zone (300 °C/2 s^−1^), (**c**) maximum power dissipation zone (450 °C/0.02 s^−1^).

Figure 12 exhibits the hot processing maps of Mg-8.7Gd-4.18Y-0.42Zr alloy at strains 0.1, 0.3, 0.5, 0.7, and 0.9, respectively, which are gained by superimposing the power dissipation diagram and the instability diagram. In these maps, the contour line values represent the power dissipation values (*η*), and the shaded domains represent the unstable deformation under different strains. Comparing the hot processing map under different strain levels. It can be found that the region with larger power dissipation efficiency (*η*) is almost concentrated in a relatively fixed range that is a low strain rate and high deformation temperature. There is no obvious change in the safety deformation domain, resulting in some slight changes in the corresponding compression temperature and strain rate. Figure 12e shows the hot processing map with the strain of 0.9, in which the values of power dissipation efficiency under different deformation conditions are given and divided into two regimes. The first state indicates that the power dissipation efficiency (*η*) of the studied alloy is less than 0.1, the corresponding deformation condition is in the range of 300–350 °C, and the strain rate is higher than 0.2 s^−1^ shown by the red symbol in Figure 12e. Figure 13b shows the microstructure images of the instability zone (300 °C/2 s^−1^). It is found that there is no clear fine grain except for the micro-crack caused by the unstable process, visible under a low magnification of 200×. The phenomenon of flow instability is caused by the initiation and propagation of microcracks, which is attributed to the failure of heat generated by plastic deformation to transfer to the surrounding area in time. For Mg alloy with an HCP crystal structure, the prismatic and pyramidal slip cannot be fully activated at a low deformation temperature due to does not satisfy the slip rule. With the increase of strain, the slip directions of adjacent grains are quite different, and dislocations are effectively blocked at the interface of the grains. Then the accumulation of dislocations at grain boundaries indicates that there may be strong plastic strain incompatibility and high stress concentration near grain boundaries. Therefore, when the distortion degree of a certain part exceeds the critical value, the interface crack begins to nucleate. The second state represents that the energy dissipation efficiency (*η*) of the alloy is greater than 0.4, and the compression temperature of 400–475 °C with the strain rate is lower than 0.02 s^−1^ shown by green symbol. For the metals with low stacking fault energy, the maximum power dissipation efficiency of DRX is approximately 0.35, such as magnesium alloy. Nevertheless, it is about 0.5 for metals with high stacking fault energy [21]. The optical microstructure of the alloy deformed under these deformation conditions is shown in Figure 13c, corresponding to the red marked area in Figure 12e. There is a large number of fine DRX grains in initial grain boundaries. The hot deformation microstructure of Mg-8.7Gd-4.18Y-0.42Zr magnesium alloy gradually changed into a fully dynamic recrystallization microstructure with the increase of energy dissipation efficiency (*η*). However, the nucleation of dynamic recrystallization grains may occur at distorted grain boundaries, deformation bands, and twining grain boundaries with higher energy. Therefore, the flow instability region and safe deformation region can be determined by a hot processing map. However, it is necessary to undertake further detailed observation of the metallographic microstructure to explain, verify and confirm the mechanism, which will be discussed in the following sections.

### 3.5. Microstructure Evolution

**Figure 14:**
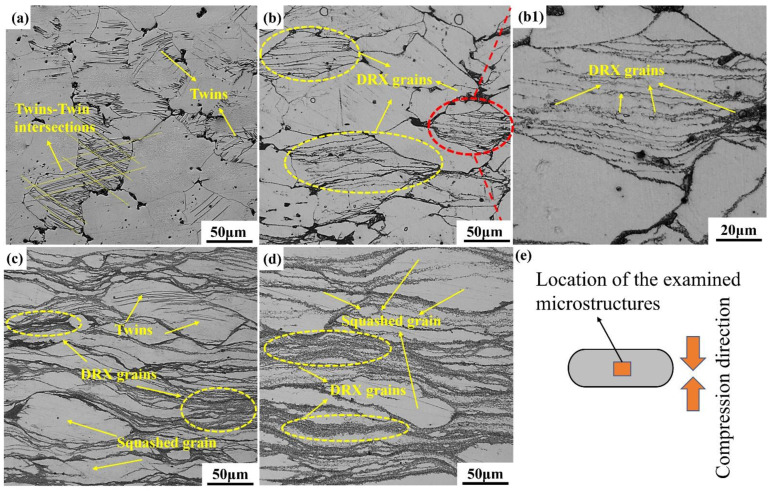
Microstructure of compressed samples with strain rate is 0.02 s^−1^ at 300 °C: (**a**) *ε* = 0.1, (**b**) *ε* = 0.3, (**c**) *ε* = 0.7, (**d**) *ε* = 0.9, (**b1**) The bigger magnification microstructure of (**b**), (**e**) Schematic diagram of the location of the examined microstructures.

Figure 14 shows the optical metallographic microstructure of the specimens under different strain conditions at 300 °C. The location of the examined microstructure and the schematic of compression direction is shown in Figure 14e. With the strain of 0.1, a great number of lenticular and intersecting twins were observed in the original grains. The angle between twins and compression direction was about 45°. When the strain rate was 0.3, the nucleation sites of DRX were transferred from the twin intersection towards the grain interior, and the necklace-like structures composed of fine DRX grains were formed. The dynamically recrystallized grains in the original grains by the bigger magnification are clearly observed in Figure 14(b1). Further increasing the compressive strain of 0.7. It can be observed that there were a great number of DRX grains in the grain and along the grain boundaries. The phenomenon of twin–twin intersections disappeared and there were some lenticular twins that appeared in the grains. With the strain increased to 0.9, the volume fraction of recrystallized grains increased with increasing strain. However, the microstructure of the alloy was still inhomogeneous, consisting of squashed grains and fine recrystallized grains, as shown in Figure 14.

**Figure 15:**
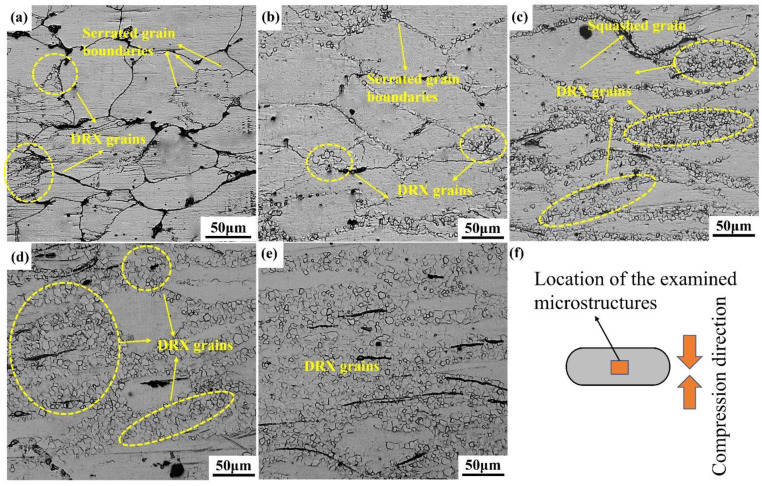
Microstructure of compressed samples with strain rate is 0.02 s^−1^ at 400 °C: (**a**) *ε* = 0.1, (**b**) *ε* = 0.3, (**c**) *ε* = 0.5, (**d**) *ε* = 0.7, (**e**) *ε* = 0.9, (**f**) Schematic diagram of the location of the examined microstructures.

Figure 15 shows the microstructure of samples with different strains at 400 °C and 0.02 s^−1^, which correspond to specimens gained near the relatively higher efficiency conditions. When the compressive strain was 0.1, it was clearly observed that the original grain boundary exhibited the phenomenon of bowing out, demonstrating serrated grain boundary characteristics. Meanwhile, there were some DRX grains appearing in the vicinity of the initial grain boundaries. Further increasing the strain to 0.3, it can be seen from Figure 15b that many fine DRX grains exist along the initial grain boundaries and at the grain boundary triple junctions, while the serrated grain boundaries were observed. Ponge et al. [38] have shown that the fluctuation of grain boundary growth shape is the main reason for the initiation of DRX. Further increasing strain, the volume fraction DRX increased and expanded towards the inner of the initial grains. On the one hand, with the increase in deformation temperature, the kinetic energy between atoms increases, and more and more slip systems were opened, which led to the development of deformation in a favorable direction. On the other hand, with the increase of slip systems, the probability of dislocation interlacing in each slip system increased and led to the rapid increase of energy storage in the alloy, which provided favorable conditions for the occurrence of dynamic recrystallization in the later stage. It is well known that the dynamic recrystallization process can be divided into continuous dynamic recrystallization (CDRX) and discontinuous dynamic recrystallization (DDRX). The continuous dynamic recrystallization is characterized by deformation-induced dislocation accumulation, entanglement, dislocation wall, and dislocation cell formation. With the increase of strain, low angle grain boundaries (LAGBs 2–15°) gradually transform into large angle grain boundaries (LAGBs > 15°) [39]. Discontinuous dynamic recrystallization is a classical type of dynamic recrystallization, which mainly occurs during the thermal deformation of low-stacking fault energy metals. It is characterized by the nucleation of new grains by bowing out of initial grain boundaries, local migration, accumulation, and rearrangement of internal dislocations, then the growth of newly nucleated recrystallized grains through migration of the grain boundaries. Finally, leading to all the un-recrystallized grains are consumed completely. Based on what has been discussed above, it is concluded that the mechanism of dynamic recrystallization is discontinuous dynamic recrystallization. However, From the true stress–strain curve shown in Figure 3a–c, which shows that the peak stress is almost equal to the steady-state stress during steady-state flow, it is concluded that the recrystallization mechanism is considered to be continuous dynamic recrystallization [40].

**Figure 16:**
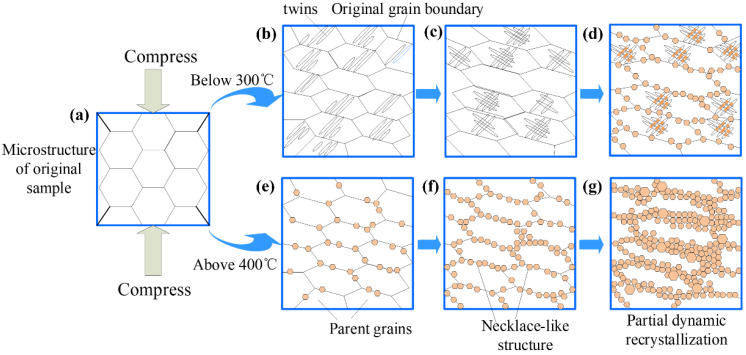
Schematic showing the mechanism of dynamic recrystallization during continuous compression deformation. (**a**) the microstructure of the as-solutioned specimen, (**b**)the formation of lenticular twins, (**c**) twin–twin intersections, (**d**) the DRX grains nucleate at the initial grain boundaries and expand outward. (**e**) DRX grains nucleating at the original grain boundary, (**f**) the formation of the necklace-like structures, (**g**) DRX grains expand inward the original grains.

The dynamic recrystallization mechanism of Mg-8.7Gd-4.18Y-0.42Zr magnesium alloy under different deformation conditions is expounded, as shown in Figure 16. The microstructure of the original specimen is composed of equiaxed grains. When the compression temperature is below 300 °C, the microstructure starts with the formation of lenticular twins, as shown in Figure 16b, while the dislocations are also activated [41]. With the increase of strain, the phenomenon of twin–twin intersections commences appearing, in which DRX grains gradually nucleate and grow up. Then the volume fraction of DRX grains gradually increased and began to expand outward with further increasing strain, while the DRX grains nucleate and grow up along the initial grain boundaries, as shown in Figure 16d. Compared with the compression temperature above 400 °C, DRX grains proceed from nucleating at the original grain boundary at the early deformation stage. Supposing dynamic recrystallization results in grain refinement, the necklace-like typical recrystallized structures are generally observed. With further increase of strain, DRX grains gradually expand inward the original grains shown in Figure 16g. Therefore, When the compression temperature is above 400 °C, DRX grains nucleate preferentially at the original grain boundary, Then gradually expand inward with the increase of strain. Nevertheless, Under the deformation conditions below 300 °C, the DRX grains nucleate preferentially at the twin–twin intersections in the parent grains and gradually expand outward with the increase of strain.

## 4. Conclusions

Based on the thermal simulation results of Mg-8.7Gd-4.18Y-0.42Zr alloy, the sensitivity of flow stress to deformation temperature and strain rate were studied. At the initial stage of deformation, the flow stress increases rapidly and reaches the maximum with the increase of strain rate due to work hardening. Then, the flow stress decreases slowly. When the strain exceeds a certain value, the flow stress gradually tends to a relatively stable state. The flow behavior of the studied alloy in the strain rate range of 0.002–10 s^−1^ is as follows:

(1) The flow stress of Mg-8.7Gd-4.18Y-0.42Zr alloy increases with the decrease of deformation temperature and the increase of strain rate due to the interaction of work hardening and dynamic softening.
(2) The average deformation activation energy and the constitutive equation of the Mg-8.7Gd-4.18Y-0.42Zr alloy by the Arrhenius model are obtained as 227.67 KJ/mol and $\dot{\varepsilon }=1.59\times 10^{16}[\sinh{\left(0.009\sigma \right)}^{4.34}\exp(-227.67/RT)$.
(3) The softening mechanism of Mg-8.7Gd-4.18Y-0.42Zr alloy is mainly dynamic recrystallization, which can be obtained from the phenomenon that the flow stress decreases after reaching the peak value and the microstructure characteristics of the compressive specimens under various deformation conditions. When the compression temperature of 400 °C, DRX grains nucleate preferentially at the original grain boundary, then gradually expand inward with the increase of strain. Nevertheless, Under the condition of 300 °Ccompression, the DRX grains nucleated preferentially at the twin–twin intersections in the parent grains and gradually expand outward with the increase of strain.
(4) The optimum deformation parameters of Mg-8.7Gd-4.18Y-0.42Zr alloy are determined as a temperature of 400–450 °C and strain rate of 0.002–0.02 s^−1^ by comprehensive comparison of hot process map, hot workability, and microstructure refinement.

## Figures and Tables

**Table 1:** The failure strains of the specimens deformed at the low temperature of 300 °C and 350 °C.

	0.002 s^−1^	0.02 s^−1^	0.2 s^−1^	2 s^−1^	10 s^−1^
300 °C	/	/	0.32	0.30	0.23
350 °C	/	/	0.63	/	/

**Table 2:** Critical strains (*ε_c_*), peak strain (*ε_p_*), and their ratio (*ε_c_*/*ε_p_*) at different compression conditions.

Temperature/°C	$\dot{\varepsilon }\;$= 0.02 s^−1^			$\dot{\varepsilon }\;$= 0.2 s^−1^			$\dot{\varepsilon }\;$= 2 s^−1^			$\dot{\varepsilon }\;$= 10 s^−1^		
	$\varepsilon_{c}$	$\varepsilon_{p}$	$\varepsilon_{c}$ $/\varepsilon_{p}$	$\varepsilon_{c}$	$\varepsilon_{p}$	$\varepsilon_{c}$ $/\varepsilon_{p}$	$\varepsilon_{c}$	$\varepsilon_{p}$	$\varepsilon_{c}$ $/\varepsilon_{p}$	$\varepsilon_{c}$	$\varepsilon_{p}$	$\varepsilon_{c}$ $/\varepsilon_{p}$
300 °C	0.204	0.277	0.73	0.277	0.329	0.84	0.226	0.298	0.75	0.199	0.232	0.85
350 °C	0.166	0.214	0.77	0.228	0.27	0.84	0.185	0.248	0.74	0.186	0.227	0.81
400 °C	0.028	0.036	0.77	0.184	0.262	0.70	0.14	0.204	0.68	0.172	0.201	0.85
450 °C	0.024	0.033	0.72	0.027	0.039	0.69	0.143	0.189	0.75	0.144	0.186	0.77
475 °C	0.015	0.025	0.72	0.018	0.027	0.66	0.119	0.177	0.67	0.12	0.171	0.70

## Data Availability

Not applicable.
